# Cancer Risk in Clinically Recognized Celiac Disease: A Nationwide Propensity-Matched Cohort Study

**DOI:** 10.3390/medsci14030352

**Published:** 2026-06-27

**Authors:** Reem Zabit, Ahmad Shibly, Jamal Zidan, Ofir Cohen, Ismaell Massalha

**Affiliations:** 1 Department of Pediatrics, Ziv Medical Center, Safed 1300000, Israel; 2 Clalit Health Services Community Clinic, Karmey Gat 8202280, Israel; 3 Department of Oncology, Ziv Medical Center, Safed 1300000, Israel; 4 Faculty of Computer and Information Science, Ben-Gurion University of the Negev, Beer Sheva 8410501, Israel; 5 Faculty of Health Sciences, Ben-Gurion University, Beer Sheva 8410501, Israel; 6 Department of Radiation Oncology, Ziv Medical Center, Safed 1300000, Israel

**Keywords:** celiac disease, malignancy, lymphoma, gastrointestinal cancer, electronic health records, propensity score matching, hematologic malignancy, observational cohort

## Abstract

**Background/Objectives**: Celiac disease (CD) is common, but its cancer-risk profile remains incompletely defined. Estimates vary because of referral patterns, diagnostic era, outcome definitions, and surveillance around diagnosis. We evaluated cancer-category-specific associations in a matched cohort of clinically recognized CD. **Methods**: We used longitudinal electronic health record (EHR) data from Clalit Health Services for a propensity-matched cohort. Adults with EHR-coded CD were matched to controls on demographic, socioeconomic, comorbidity, and inflammatory variables. Pre-index invasive malignancies and non-invasive neoplasms were excluded. Dated EHR-coded invasive oncology outcomes were analyzed using Cox models. A restricted dated-event cohort, lag analyses, competing-risk modeling, hemoglobin adjustment, and age-at-index strata assessed robustness. **Results**: The primary matched cohort included 8143 individuals: 1006 with CD and 7137 controls, contributing 49,330.5 person-years. CD was associated with increased hazard of an EHR-coded invasive oncology outcome (hazard ratio [HR] 1.61, 95% confidence interval [CI] 1.47–1.77; p<0.001). Strongest signals were hematological malignancy codes (HR 1.99), lymphoma codes (HR 1.90), and gastrointestinal (GI) cancer codes (HR 2.71). Associations persisted after one-year and two-year lags. In the dated-event sensitivity cohort (161 CD; 1610 controls), CD remained associated with invasive cancer (HR 1.68, 95% CI 1.31–2.14), with the strongest signals for lymphoma (HR 2.81) and GI cancer (HR 2.25). The association was essentially unchanged under competing-risk modeling (Fine–Gray subdistribution HR 1.69) and after hemoglobin adjustment (HR 1.61), and was present in both age strata. Neither breast nor lung cancer was associated. Lymphoma codes included peripheral T-cell lymphomas recorded at intra-abdominal and extranodal sites, the pattern most consistent with enteropathy-associated T-cell lymphoma (EATL). **Conclusions:** In clinically recognized CD, cancer hazard was elevated and category-specific, concentrated in hematological, lymphoid, and GI codes with a gut-oriented T-cell lymphoma signal. The findings support targeted clinical vigilance, not expanded screening, and describe relative associations that require registry-linked confirmation.

## 1. Introduction

The therapeutic landscape of oncology has shifted toward molecularly targeted agents, immunotherapies, and risk-stratified care pathways. Those approaches are most useful when the upstream risk signal is specific. Chronic immune-mediated diseases are a natural place to ask that question, because persistent antigenic stimulation, epithelial injury, and lymphoid activation may converge long before a cancer diagnosis. Celiac disease (CD) belongs in that group: common, biologically active, and historically linked to malignancy, but not in a uniform way.

CD affects approximately 1% of many populations and is now recognized across a broad adult clinical spectrum [1,2,3]. Wider serological testing has moved the field away from the older picture of severe malabsorptive disease diagnosed late. A modern clinical cohort therefore captures a mixture of symptomatic patients, screen-detected disease, variable disease duration before diagnosis, and variable response to a gluten-free diet. Cancer estimates from older referral cohorts cannot simply be carried forward.

Early diagnosis and sustained gluten-free diet adherence remain central to reducing preventable complications of CD [4,5]. This matters for oncology because untreated or poorly controlled enteropathy may prolong mucosal injury, nutritional depletion, and immune activation. Diet alone, however, does not fully explain the malignancy signal. Lymphoproliferative complications, particularly in refractory phenotypes, can emerge despite reported dietary adherence, which separates modifiable inflammatory exposure from intrinsic or acquired lymphoid risk [6,7].

The most consistent malignancy signals in CD have not been “all cancer” signals. Meta-analytic and nationwide data point toward a modest overall increase, with stronger or more persistent associations for hematological, lymphoproliferative, and selected gastrointestinal (GI) malignancies [8,9,10]. Small bowel adenocarcinoma and enteropathy-associated T-cell lymphoma (EATL) remain uncommon, but they are central to the clinical concern because they are biologically plausible and clinically consequential [11,12].

Mechanistic work supports this selective pattern. Chronic mucosal inflammation, intraepithelial lymphocyte activation, interleukin-15 signaling, and somatic alterations involving JAK1-STAT3 and NF-κB pathways provide a credible route from uncontrolled enteropathy to lymphomagenesis [13,14,15]. Refractory celiac disease (RCD) is the clinical bridge where this biology becomes most visible: type I RCD retains a largely normal intraepithelial lymphocyte phenotype, whereas type II RCD (RCDII) is marked by aberrant clonal intraepithelial lymphocyte expansion and carries substantial risk of progression to EATL in specialized cohorts [7,16]. The same biology does not imply a generalized increase across every solid tumor site. That distinction matters for patient counseling.

Epidemiologic interpretation is still difficult. Studies differ in CD ascertainment, use of biopsy confirmation, outcome source, handling of prevalent cancers, inclusion of non-invasive neoplasms, lag periods, and the degree to which healthcare utilization is measured. Electronic health record (EHR)-coded oncology outcomes add another layer: they can support large-scale relative-risk analyses, but registry linkage is needed for calibrated absolute incidence and first-primary cancer definitions [17].

We aimed to estimate relative associations between clinically recognized CD and dated invasive oncology outcomes within EHR-coded follow-up in a large temporally aligned matched cohort. The analysis was designed to identify reproducible cancer-category patterns, supported by lag-time analyses and a restricted dated-event sensitivity cohort, rather than to derive population absolute incidence rates.

## 2. Methods

### 2.1. Data Source and Study Design

We conducted a retrospective, population-based matched cohort study using longitudinal electronic health record data from Clalit Health Services, the largest healthcare provider in Israel, covering approximately 52% of the national population. The database includes demographic characteristics, clinical diagnoses, laboratory measurements, and mortality data, with longitudinal follow-up.

The study protocol was approved by the Soroka University Medical Center, a Clalit Health Services hospital, institutional Helsinki Committee (approval no. SOR-0184-24) on 14 August 2024, with a waiver of informed consent due to the retrospective and fully de-identified nature of the data, which were drawn from Clalit Health Services electronic health records. This study is reported in accordance with the Strengthening the Reporting of Observational Studies in Epidemiology guideline for cohort studies.

### 2.2. Study Population

Adult patients (≥18 years) with a documented diagnosis of CD were identified using structured clinical fields. The index date for CD patients was defined as the earliest available celiac diagnosis date and was reconstructed using diagnosis codes, reference event dates, and age-at-diagnosis fields where applicable. Because CD ascertainment relied on documented diagnoses in the electronic record, the exposure definition reflects clinically recognized CD rather than undiagnosed disease in the community. The coded CD phenotype was not validated in this analysis against tissue transglutaminase IgA, endomysial antibody, or duodenal biopsy results; it should therefore be interpreted as an operational EHR-coded clinical phenotype. Non-differential misclassification of this phenotype would be expected to attenuate associations toward the null, whereas more intensive coding and follow-up among clinically complex patients could inflate apparent excess risk. To probe the latter, we defined a high-specificity exposure requiring at least two independent CD documentation fields (Section 2.5).

Control individuals without CD were selected from the same source population. All participants were required to be alive and free of invasive malignancy at the assigned index date. Prevalent invasive cancers and non-invasive neoplasms, including carcinoma in situ, diagnosed at or before the index date were excluded.

### 2.3. Ascertainment of Malignancy Outcomes

Dated invasive oncology outcomes were identified from oncology diagnosis records and curated oncology event fields, and are referred to throughout as cancer outcomes. Each diagnosis required a valid date strictly after the index date. Non-invasive neoplasms, including carcinoma in situ and benign tumors, were excluded. For each endpoint, follow-up ended at the first qualifying post-index record. These outcomes were EHR-coded and were not validated against Israeli National Cancer Registry first-primary morphology records.

Hematological malignancies were classified as leukemia or lymphoma based on diagnosis descriptions and oncology disease groupings. Solid tumor subtypes, including gastrointestinal, breast, and lung cancers, were identified using diagnosis-specific text patterns and oncology classification fields. Within the lymphoma category, diagnosis descriptions denoting peripheral T-cell lymphoma were flagged, and those recorded at intra-abdominal or extranodal and solid-organ sites were treated as an EHR-coded proxy for EATL; these were examined descriptively and not relabeled as histologically confirmed EATL. Patients with cancer-related diagnosis codes lacking a valid diagnosis date were excluded from time-to-event analyses to preserve temporal validity. Additional processing and subtype-detection summaries are provided in Supplementary Tables S5 and S6.

### 2.4. Covariates and Matching

Baseline covariates included age at index date, sex, socioeconomic status (SES), Charlson Comorbidity Index (CCI), and systemic inflammatory status measured by neutrophil-to-lymphocyte ratio (NLR). Laboratory values were derived from measurements closest to, but preceding, the index date. Missing or implausible NLR values were imputed using cohort-specific medians.

CD patients were matched 1:10 to non-celiac controls using nearest-neighbor propensity score matching without replacement. The propensity score model included age, sex, SES, CCI score, and NLR. Balance was assessed using standardized mean differences (SMDs), with values < 0.10 generally indicating adequate balance. For matched controls, the index date was assigned as the index date of the corresponding CD patient within each matched subclass.

### 2.5. Sensitivity Analyses

To address potential reverse causality and early diagnostic clustering, 1-year and 2-year lag-time sensitivity analyses were performed in the primary cohort by excluding events occurring within the lag window and shifting the time origin accordingly.

We also repeated the principal Cox models in a restricted dated-event sensitivity cohort requiring complete oncology-event timing. Because this restriction was intended to strengthen endpoint confirmation rather than define the source-population denominator, the analysis focused on relative associations rather than absolute incidence rates and used the same core adjustment set as the primary models. Within this dated-event cohort we ran four additional analyses: (i) a hemoglobin-adjusted Cox model, since hemoglobin was not a matching variable and differed at baseline; (ii) a Fine–Gray competing-risk model treating death as a competing event; (iii) models stratified by age at index date, dichotomized at the cohort median, as a coarse proxy for earlier versus later recognition; and (iv) a high-specificity exposure analysis restricted to CD patients with at least two independent CD documentation fields.

### 2.6. Statistical Analysis

Time-to-event was calculated from the index date to the first qualifying cancer outcome or censoring at death or administrative end of follow-up (20 December 2025), whichever occurred first. Cox proportional hazards regression models were fitted to estimate hazard ratios (HRs) and 95% confidence intervals (CIs), with robust standard errors clustered by matched subclass to account for within-set correlation. Proportional hazards assumptions were assessed using Schoenfeld residuals. Death was additionally modeled as a competing risk using the Fine–Gray subdistribution hazard approach, yielding subdistribution hazard ratios (SHRs). Group differences in subtype-specific diagnosis-code counts, including the T-cell and EATL-proxy categories, were tested with Fisher’s exact test.

Primary analyses evaluated the association between CD and any cancer outcome. Secondary analyses examined hematological malignancies, lymphoma, leukemia, and selected solid tumor subtypes. Lag-time, restricted-endpoint, competing-risk, hemoglobin-adjusted, age-stratified, and high-specificity analyses were used to evaluate the stability of the principal association under alternative temporal, endpoint, and exposure definitions. All analyses were conducted using R (version 4.2.3), with statistical significance defined as a two-sided *p*-value < 0.05.

## 3. Results

### 3.1. Primary Cohort and Baseline Characteristics

After cohort construction (Supplementary Figure S1), the primary matched cohort comprised 8143 individuals, including 1006 patients with CD and 7137 matched controls. Balance across prespecified matching variables was achieved following propensity score matching (Table 1; Supplementary Figure S2). Laboratory variables shown for descriptive purposes, such as hemoglobin, were not included in the matching algorithm and therefore may exhibit residual imbalance.

The mean age at index date was 49.0 years (standard deviation [SD] 28.0) in the overall cohort, with a mean of 48.2 years (SD 26.2) among patients with CD and 49.1 years (SD 28.2) among controls. Women accounted for 47.1% of participants overall, including 46.0% in the CD group and 47.3% among controls. Comorbidity burden and baseline inflammatory markers were comparable between groups.

### 3.2. Primary Cox Analysis

The primary matched cohort contributed 49,330.5 person-years (PY) of follow-up, including 4212.8 PY among patients with CD and 45,117.7 PY among matched controls. Because oncology outcomes were derived from EHR-coded diagnosis/event fields rather than registry-confirmed first-primary invasive cancer records, absolute crude rates were not used as inferential targets. The analysis therefore focused on relative hazard estimates within the matched cohort.

In Cox regression adjusted for sex, SES, CCI, and NLR, CD was associated with increased hazard of a cancer outcome (HR 1.61, 95% CI 1.47–1.77; p<0.001; Table 2). Very low SES and higher CCI score were independently associated with higher outcome hazard.

**Figure 1:**
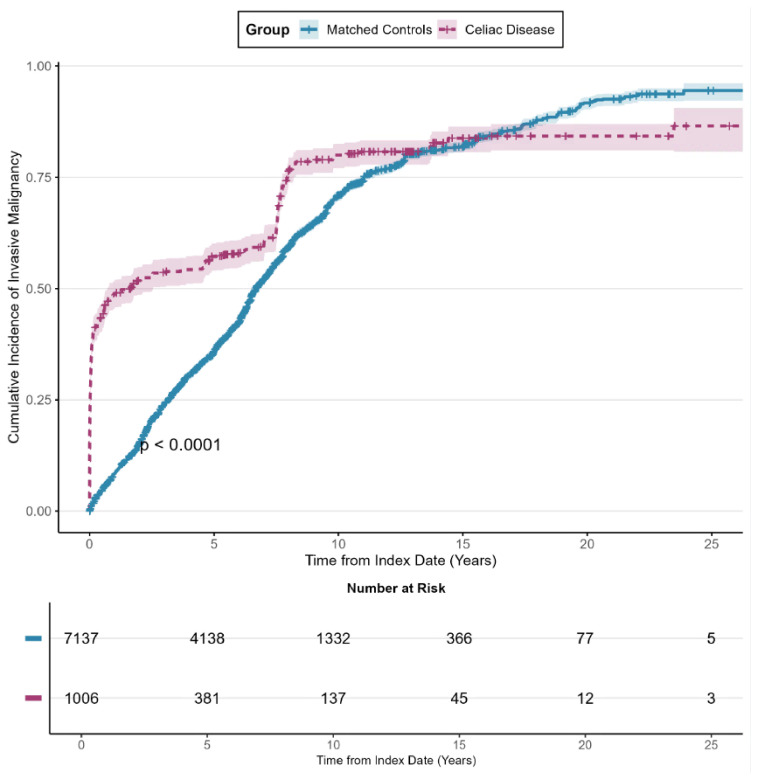
Cumulative occurrence of cancer outcomes (1 – Kaplan–Meier estimate; death-censored) in patients with celiac disease and matched controls, primary cohort.

Kaplan–Meier curves demonstrated separation between groups, with higher cumulative occurrence of cancer outcomes among patients with CD (log-rank p<0.001; Figure 1).

### 3.3. Cancer Subtypes in the Primary Cohort

The association was strongest for the hematological categories. CD was associated with a two-fold increased hazard of hematological malignancy codes (HR 1.99, 95% CI 1.75–2.26; p<0.001) and a 1.9-fold increased hazard of lymphoma codes (HR 1.90, 95% CI 1.61–2.25; p<0.001; Table 3; Supplementary Tables S1 and S2). Leukemia alone was not statistically significant (HR 0.88, 95% CI 0.75–1.02; p=0.091).

Among solid tumor categories, CD was associated with increased hazard of GI cancer codes (HR 2.71, 95% CI 2.10–3.49; p<0.001). An inverse association was observed for breast cancer (HR 0.48, 95% CI 0.24–0.96; p=0.037), though event counts were modest and this finding was considered exploratory. No significant association was observed for lung cancer (HR 1.06, 95% CI 0.69–1.64; p=0.777).

**Figure 2:**
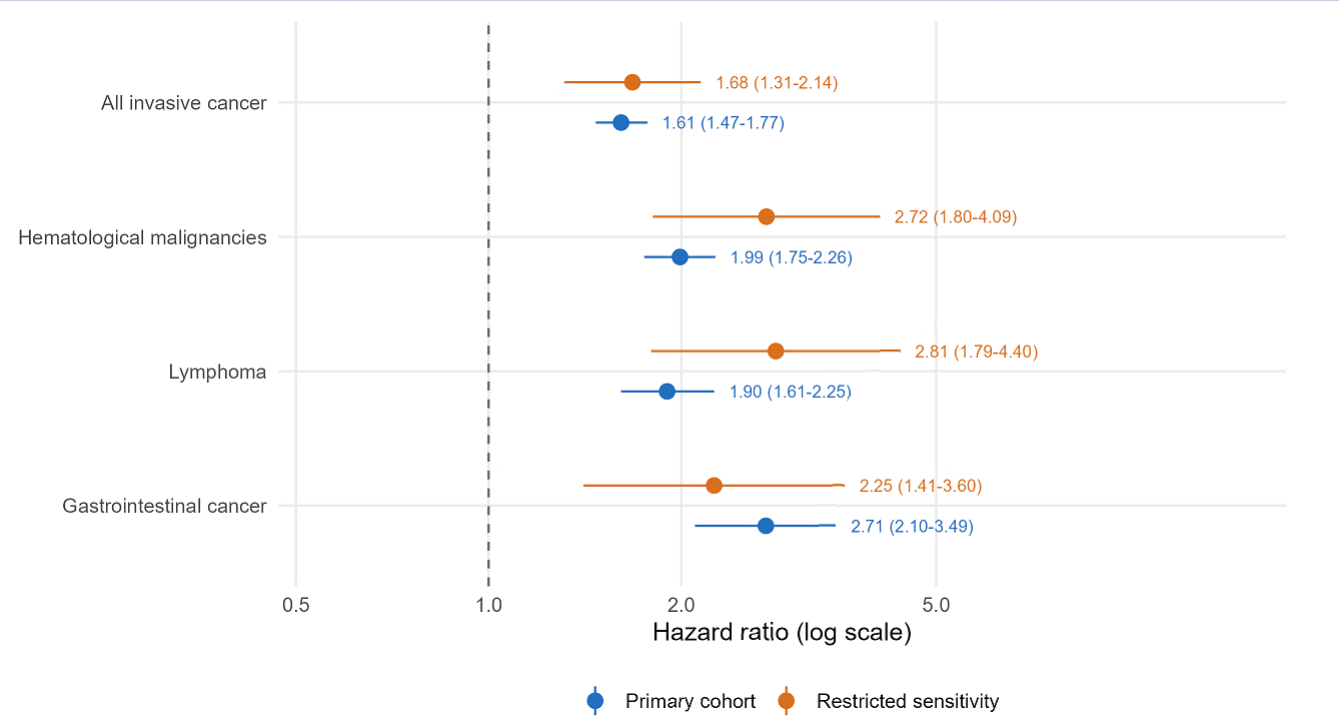
Adjusted hazard ratios for cancer-outcome categories in celiac disease: primary and dated-event sensitivity cohorts.

A summary of primary and restricted sensitivity hazard ratios across malignancy categories is shown in Figure 2.

### 3.4. Lag-Time and Dated-Event Sensitivity Analyses

After applying 1-year and 2-year lag periods in the primary cohort, the association between CD and cancer outcomes remained significant (Table 4; Supplementary Tables S3 and S4). All estimates in Table 4 are presented as CD versus matched controls.

The dated-event sensitivity cohort comprised 161 patients with CD and 1610 propensity-matched controls (1771 individuals). It reproduced the principal relative pattern: CD was associated with invasive cancer (HR 1.68, 95% CI 1.31–2.14; p<0.001), with the strongest signals for lymphoma and GI cancer (Table 5). Leukemia, which was not significant in the primary cohort, reached significance in this dated-event analysis. Neither breast nor lung cancer was associated. Per-category diagnosis-record counts behind these subtype models are listed in Supplementary Table S6; the smaller endpoints (leukemia, breast, and lung) rest on few events, so we read them as exploratory.

### 3.5. Competing-Risk, Hemoglobin-Adjusted, Age-Stratified, and High-Specificity Analyses

The dated-event association was stable across the robustness analyses requested in review (Table 6; Supplementary Tables S7–S10). Modeling death as a competing event produced a Fine–Gray subdistribution HR of 1.69 (95% CI 1.31–2.19; p<0.001), essentially identical to the cause-specific estimate, so competing mortality does not explain the finding. Adding hemoglobin to the model left the celiac estimate at HR 1.61 (95% CI 1.26–2.05; p<0.001), with lower hemoglobin independently associated with higher hazard (HR 0.94 per g/dL, 95% CI 0.91–0.98). Stratifying by age at index showed the association in both strata and stronger in older patients (younger HR 1.37; older HR 2.12). Restricting the exposure to CD patients with at least two independent documentation fields did not change the estimate (HR 1.68); all 161 CD patients met this criterion.

### 3.6. T-Cell Lymphoma and EATL-Proxy Codes

Histological subtyping is not available in the EHR, but the diagnosis-level descriptions within the lymphoma category were not generic. They included peripheral T-cell lymphomas recorded at intra-abdominal lymph nodes and at extranodal and solid-organ sites, the distribution most consistent with EATL (Supplementary Table S11). Peripheral T-cell lymphoma codes were recorded in 4 of 161 CD patients versus 2 of 1,610 controls (Fisher exact odds ratio [OR] 20.4, 95% CI 2.9–226.6; p=0.0009), and the gut-oriented EATL-proxy pattern in 4 CD patients versus 1 control (OR 40.8, 95% CI 4.0–1994; p=0.0003; Table 7). These counts are small and the confidence intervals are correspondingly wide; the comparison is descriptive and exploratory, and the codes were not relabeled as histologically confirmed EATL.

## 4. Discussion

In this population-based matched cohort, clinically recognized CD carried a higher hazard of cancer outcomes. The result was not a diffuse cancer signal. Excess relative hazard clustered in hematological malignancy codes, lymphoma codes, and GI cancer codes, while lung cancer was not increased. The dated-event sensitivity cohort reproduced the same principal pattern despite a much smaller analytic sample, and the association held when death was modeled as a competing risk and when hemoglobin was added to the model.

The pattern agrees with the direction of prior evidence, although the magnitude is design-specific. Meta-analytic data have described a modest overall malignancy excess in CD, and a large Swedish registry cohort found that the overall cancer increase was small, concentrated near the time of diagnosis, and driven mainly by selected hematological and GI categories [8,9]. A recent mortality meta-analysis similarly found that malignancy-related mortality is elevated in CD, with non-Hodgkin lymphoma carrying the clearest excess mortality signal [10]. Our cohort differs by using a clinically recognized EHR-coded exposure and EHR-coded oncology endpoints, so what this study contributes is the relative category pattern, not a population incidence estimate.

The hematological and lymphoid findings are clinically coherent. Chronic gluten-driven inflammation, epithelial stress, and dysregulated intraepithelial lymphocyte survival provide a plausible bridge from enteropathy to lymphomagenesis. Molecular studies in refractory CD and EATL implicate JAK1-STAT3 and NF-κB pathway alterations, with interleukin-15-dependent lymphocyte expansion providing an experimental link between intestinal immune activation and lymphoma development [14,15]. Sequencing studies in RCDII and EATL have quantified these drivers. Roughly 80–90% of cases carry somatic gain-of-function JAK1–STAT3 mutations, with a recurrent JAK1 p.G1097 hotspot in about half, often alongside disruption of NF-κB regulators such as TNFAIP3 and recurrent mutations in epigenetic modifiers (TET2, KMT2D, DDX3X) [15]. IL-15 overexpression in the celiac epithelium sustains intraepithelial lymphocyte survival through JAK3–STAT5 and antiapoptotic Bcl-2/Bcl-xL signaling, the experimental bridge from a cytokine-rich mucosa to clonal selection [18]. Histological lymphoma subtypes are not in the source data, so we do not claim confirmed EATL. The diagnosis-level descriptions are nonetheless informative. The peripheral T-cell lymphomas here were recorded at intra-abdominal and extranodal sites, almost entirely on the celiac side. That is the distribution the EATL hypothesis predicts. We read it as a CD-associated lymphoproliferative phenotype, not a nonspecific excess of cancer. It rests on few events, and only registry-linked histology will settle it. For context, EATL is defined immunophenotypically (CD3ε+, CD7+, CD103+, cytotoxic markers; CD8−/CD56−, which separates it from monomorphic epitheliotropic intestinal T-cell lymphoma), and EHR diagnosis codes cannot establish that profile. This is exactly why we treat the T-cell pattern as a proxy rather than a diagnosis [4].

The GI cancer association fits the same selective-risk framework. Swedish histopathology-linked data have shown increased risk of small bowel adenocarcinoma and adenomas in CD, while broader GI estimates vary across studies because of rarity, diagnostic era, and outcome definition [11,19]. In the present analysis, the GI signal persisted after exclusion of prevalent cancers and remained visible in the dated-event cohort. That is the useful clinical message: persistent or unexplained GI symptoms in adults with CD deserve timely evaluation, especially when accompanied by nutritional decline, iron deficiency, weight loss, poor dietary response, or refractory features.

The lag-time analyses bear on timing. The all-cancer association persisted after excluding the first year and first two years of follow-up in the primary cohort (HR 1.88 and 1.89, respectively), which makes pure early detection bias an incomplete explanation. It does not remove surveillance effects. A celiac diagnosis brings more laboratory testing, endoscopy, and specialist contact, and that contact finds disease; some of the magnitude reported here reflects detection intensity rather than incidence. The Swedish registry study similarly found that excess overall cancer risk was greatest close to CD diagnosis, while selected hematological and GI signals remained clinically relevant beyond the immediate diagnostic window [9]. We therefore read surveillance as a genuine contributor to effect size, not as grounds to discard a signal that survives competing-risk modeling, hemoglobin adjustment, and stratification by age.

The inverse breast cancer association in the primary cohort should remain exploratory. It was not reproduced in the dated-event cohort. Prior explanations for lower breast cancer estimates in CD have invoked body composition, reproductive factors, screening patterns, or residual confounding; this study cannot adjudicate among them, and it should not influence clinical follow-up.

### Clinical Implications

The findings point toward clinical vigilance, not generalized cancer screening. Current CD guidelines emphasize diagnosis, dietary treatment, monitoring of response, and evaluation of persistent or refractory symptoms [2,20]. Our results fit that framework. Patients with stable, well-controlled CD should continue guideline-based care; patients with late diagnosis, prolonged diagnostic delay, severe malabsorptive presentation, persistent villous atrophy, poor dietary response, or alarm features merit earlier investigation for lymphoproliferative and GI malignancy pathways [3,7,16].

In higher-risk presentations, evaluation should remain clinically driven. Repeat duodenal biopsy with immunohistochemistry, flow cytometry, or T-cell receptor studies can help distinguish RCD subtypes and exclude occult EATL; capsule endoscopy and CT or MRI enterography are appropriate when small bowel malignancy, ulcerative jejunoileitis, or RCDII is suspected [7,16]. This is different from screening every patient. It is a lower threshold for diagnostic evaluation when the phenotype is no longer behaving like uncomplicated CD.

The categories identified here also carry different prognostic weight. EATL remains one of the most severe malignant complications of CD, and CD-associated small bowel adenocarcinoma is rare but stage-sensitive [11,12]. In absolute terms EATL is uncommon, on the order of 0.1 per 100,000 person-years and affecting roughly 0.1–3.2% of patients with CD, which is why a raised relative hazard argues for targeted vigilance rather than population screening [4,5]. A recent mortality meta-analysis found elevated malignancy-related mortality in CD, with non-Hodgkin lymphoma carrying the clearest excess signal [10]. The point is not the association itself, but earlier recognition of the patients in whom delay matters most.

## 5. Strengths and Limitations

A major strength of this study is its population-based design, large primary denominator, temporal alignment of exposure and outcome, and exclusion of prevalent cancer codes at index. Excluding carcinoma in situ and benign neoplasms reduced inflation from non-invasive lesions. Propensity score matching across demographic, socioeconomic, comorbidity, and inflammatory variables improved comparability between groups. The dated-event sensitivity cohort, together with the competing-risk, hemoglobin-adjusted, age-stratified, and high-specificity analyses, provides convergent evidence that the principal signal was not an artifact of a single endpoint, covariate, or exposure definition.

The principal limitation is ascertainment: CD and cancer were identified from structured EHR codes, not serology, biopsy, or cancer-registry linkage. Estimates are therefore relative hazards within a clinically recognized, dated-event cohort, not population incidence. Two features bound this. Non-differential misclassification would bias toward the null, and the association was unchanged under a high-specificity exposure definition; and because the index date was the first CD code, with only post-index cancers counted and prevalent cancers excluded, prevalent-case and immortal-time bias are limited. The signal also persisted under one- and two-year lags, competing-risk modeling, hemoglobin adjustment, and age stratification, so detection bias is better read as a contributor to magnitude than as the explanation. Registry-linked, histologically confirmed replication remains the definitive next step.

Residual confounding remains: gluten-free diet adherence, untreated disease duration, mucosal healing and villous atrophy, refractory status, nutritional status, smoking, alcohol, body-mass index, physical activity, family history, and autoimmune or metabolic comorbidity were not uniformly measured, and any of these may carry part of the association [11,21,22]. We therefore read the category pattern as hypothesis-generating, not causal. Subtype resolution was limited: the T-cell/EATL-proxy signal rests on few events, the smaller endpoints (breast, lung, and leukemia) are imprecise, and subtype analyses were not adjusted for multiplicity. These are exploratory and, like the single-system EHR design, await registry-linked histological confirmation in an independent population.

## 6. Future Directions

The next step is to link EHR-derived cohorts to cancer-registry data with histologically confirmed first-primary invasive cancers, allowing calibration of absolute incidence and subtype-specific risk, including histological confirmation of EATL. Better counting is only part of the work. Risk models should integrate CD phenotype, dietary adherence, mucosal healing, inflammatory biomarkers, autoimmune comorbidity, healthcare-utilization patterns, and emerging molecular markers such as T-cell receptor clonality, JAK-STAT pathway alterations, and IL-15 pathway activity [15,16]. Such models could identify the minority of patients most likely to benefit from intensive surveillance while keeping routine care proportionate for the larger group with controlled disease.

## 7. Conclusions

Clinically recognized CD was associated with an increased hazard of cancer outcomes in a large population-based matched cohort. The relative signal was concentrated in hematological, lymphoid, and GI cancer categories, was reproduced in a dated-event sensitivity cohort, and was robust to competing-risk modeling, hemoglobin adjustment, and age stratification. The lymphoma signal included peripheral T-cell lymphoma codes at intra-abdominal and extranodal sites consistent with EATL, an exploratory observation that requires histological confirmation.

These results support targeted clinical vigilance: careful early evaluation and continued attention to lymphoproliferative and GI malignancy among higher-risk CD patients. They describe relative associations, not causation, and should not be extended to broad cancer screening or absolute incidence prediction before registry-linked validation.

## Figures and Tables

**Table 1:** Baseline characteristics of the primary matched cohort.

Variable	Controls	Celiac Disease	SMD
	(*n* = 7137)	(*n* = 1006)	
Age at index date, mean (SD), years	49.08 (28.24)	48.24 (26.18)	0.031
Female sex, *n* (%)	3375 (47.3)	463 (46.0)	0.025
Male sex, *n* (%)	3762 (52.7)	543 (54.0)	
Socioeconomic status, *n* (%)			0.111
Very High	1245 (17.4)	138 (13.7)	
High	2003 (28.1)	253 (25.1)	
Medium	1269 (17.8)	189 (18.8)	
Low	1059 (14.8)	154 (15.3)	
Very Low	1256 (17.6)	218 (21.7)	
No data	305 (4.3)	54 (5.4)	
Charlson Comorbidity Index, mean (SD)	6.14 (3.56)	6.22 (3.31)	0.022
Hemoglobin, mean (SD), g/dL	10.71 (1.90)	10.17 (1.61)	0.308
Neutrophils, mean (SD), $\times 10^{9}$/L	6.09 (8.55)	5.69 (3.75)	0.060
Lymphocytes, mean (SD), $\times 10^{9}$/L	1.87 (6.10)	1.58 (4.84)	0.054
Neutrophil-to-lymphocyte ratio, mean (SD)	5.25 (4.90)	5.32 (4.13)	0.016

Abbreviations: SD, standard deviation; SMD, standardized mean difference. Matching targeted age, sex, socioeconomic status, Charlson Comorbidity Index, and neutrophil-to-lymphocyte ratio. Hemoglobin is shown descriptively and was not part of the matching algorithm.

**Table 2:** Multivariable Cox model for all cancer outcomes (primary cohort).

Variable	HR	95% CI	*p*-Value
Celiac disease	1.61	1.47–1.77	<0.001
Male sex	0.96	0.89–1.04	0.339
Socioeconomic status			
Low	0.97	0.81–1.17	0.784
Medium	0.84	0.73–0.97	0.016
Very Low	1.33	1.21–1.47	<0.001
Very High	0.90	0.79–1.03	0.127
No data	1.00	0.82–1.21	0.992
Charlson Comorbidity Index	1.04	1.02–1.05	<0.001
Neutrophil-to-lymphocyte ratio	0.99	0.98–1.00	0.012

Abbreviations: HR, hazard ratio; CI, confidence interval. Model adjusted for all listed covariates with robust standard errors clustered by matched subclass. Reference category for socioeconomic status was “High”.

**Table 3:** Hazard of specific cancer-outcome categories (primary cohort).

Outcome	HR	95% CI	*p*-Value
All invasive cancer	1.61	1.47–1.77	<0.001
Hematological malignancies	1.99	1.75–2.26	<0.001
Lymphoma	1.90	1.61–2.25	<0.001
Leukemia	0.88	0.75–1.02	0.091
Solid tumors			
Gastrointestinal cancer	2.71	2.10–3.49	<0.001
Breast cancer	0.48	0.24–0.96	0.037
Lung cancer	1.06	0.69–1.64	0.777

All models adjusted for sex, socioeconomic status, Charlson Comorbidity Index, and neutrophil-to-lymphocyte ratio, with robust standard errors clustered by matched subclass.

**Table 4:** Lag-time sensitivity analyses for all cancer outcomes (primary cohort).

Model	Outcomes	HR	95% CI	*p*-Value
Primary analysis, no lag	5711	1.61	1.47–1.77	<0.001
1-year lag	4623	1.88	1.69–2.09	<0.001
2-year lag	4131	1.89	1.69–2.11	<0.001

Lag analyses exclude events occurring within the specified lag period from the index date and shift the time origin accordingly. HRs are presented as CD versus matched controls.

**Table 5:** Restricted dated-event sensitivity cohort.

Outcome	HR	95% CI	*p*-Value
All invasive cancer	1.68	1.31–2.14	<0.001
Hematological malignancies			
Lymphoma	2.81	1.79–4.40	<0.001
Leukemia	2.13	1.02–4.45	0.044
Solid tumors			
Gastrointestinal cancer	2.25	1.41–3.60	<0.001
Breast cancer	0.84	0.32–2.22	0.726
Lung cancer	1.28	0.65–2.54	0.480

Dated-event cohort: 161 patients with celiac disease and 1610 matched controls. Models adjusted for sex, socioeconomic status, Charlson Comorbidity Index, and neutrophil-to-lymphocyte ratio, with robust standard errors clustered by matched subclass.

**Table 6:** Robustness analyses for celiac disease and all invasive cancer in the dated-event cohort.

Analysis	HR/SHR	95% CI	*p*-Value
Dated-event cohort, primary model	1.68	1.31–2.14	<0.001
Hemoglobin-adjusted Cox	1.61	1.26–2.05	<0.001
Fine–Gray, death as competing risk	1.69	1.31–2.19	<0.001
Age < median at index (<63.9 y)	1.37	—	—
Age ≥ median at index (≥63.9 y)	2.12	—	—
High-specificity CD (≥2 documentation fields)	1.68	—	—

Abbreviations: HR, hazard ratio; SHR, subdistribution hazard ratio; CI, confidence interval. Age-stratified and high-specificity estimates are point estimates; full models appear in Supplementary Tables S9 and S10.

**Table 7:** T-cell lymphoma and EATL-proxy diagnosis codes in the dated-event cohort.

Code Category	CD	Controls	OR (95% CI)	*p*-Value
	(*n* = 161)	(*n* = 1610)		
Peripheral T-cell lymphoma, any site	4 (2.5%)	2 (0.1%)	20.4 (2.9–226.6)	0.0009
EATL-proxy (T-cell lymphoma,	4 (2.5%)	1 (0.06%)	40.8 (4.0–1994)	0.0003
intra-abdominal/extranodal)				

Abbreviations: OR, odds ratio; CI, confidence interval; EATL, enteropathy-associated T-cell lymphoma. Odds ratios and *p*-values are from Fisher’s exact test.

## Data Availability

The data presented in this study are available on request from the corresponding author due to privacy and regulatory restrictions on Clalit Health Services electronic health record data and subject to institutional approval.

## Supplementary Materials

The following supporting information can be downloaded at: https://www.mdpi.com/article/10.3390/medsci14030352/s1. Table S1. Full multivariable Cox regression model for hematological malignancies; Table S2. Full multivariable Cox regression model for lymphoma; Table S3. Full multivariable Cox regression model for 1-year lag sensitivity analysis; Table S4. Full multivariable Cox regression model for 2-year lag sensitivity analysis; Table S5. Cancer identification and exclusion summary; Table S6. Cancer subtype detection summary from source diagnosis records; Table S7. Full multivariable Cox regression model with hemoglobin added, dated-event cohort; Table S8. Fine–Gray subdistribution hazard model with death as a competing event, datedevent cohort; Table S9. Celiac disease and all invasive cancer under a high-specificity exposure definition; Table S10. Celiac disease and all invasive cancer stratified by age at index date; Table S11. Lymphoma diagnosis descriptions classified as peripheral T-cell lymphoma or EATL-proxy; Figure S1. Cohort construction and analytic flow; Figure S2. Standardized mean differences for variables shown in the primary matched cohort baseline table. Hemoglobin was shown descriptively and was not included in the matching algorithm.

## Abbreviations

Abbreviation	Full Term
CD	Celiac disease
CCI	Charlson Comorbidity Index
CI	Confidence interval
EATL	Enteropathy-associated T-cell lymphoma
EHR	Electronic health record
GI	Gastrointestinal
HR	Hazard ratio
NLR	Neutrophil-to-lymphocyte ratio
OR	Odds ratio
PY	Person-years
RCD	Refractory celiac disease
RCDII	Refractory celiac disease type II
SD	Standard deviation
SES	Socioeconomic status
SHR	Subdistribution hazard ratio
SMD	Standardized mean difference
