# Supplementary material for: Cancer Risk in Clinically Recognized Celiac Disease: A Nationwide Propensity-Matched Cohort Study
Source: Med Sci (Basel). 2026 Jun 27;14(3):352. doi: 10.3390/medsci14030352 (PMC13413702; doi:10.3390/medsci14030352)
Supplement: Supplementary file 1 [file medsci-14-00352-s001.zip › medsci-4371529-supplementary.pdf]

Supplementary Material: Cancer Risk in Clinically Recognized Celiac Disease: A Nationwide Propensity-Matched Cohort Study

Supplementary material accompanying the manuscript by Zabit et al.

Supplementary Methods

The supplementary material provides additional model outputs, endpoint-definition checks, and summary figures supporting the primary manuscript. The primary analysis used the population-based matched cohort of 1,006 patients with celiac disease and 7,137 matched controls. Outcomes should be interpreted as dated electronic health record (EHR)-coded invasive oncology outcomes rather than registry-confirmed first-primary invasive cancers. The dated-event sensitivity cohort comprised 161 patients with celiac disease and 1,610 matched controls (1,771 individuals); full outputs are given in Tables S7–S11.

Supplementary Figures

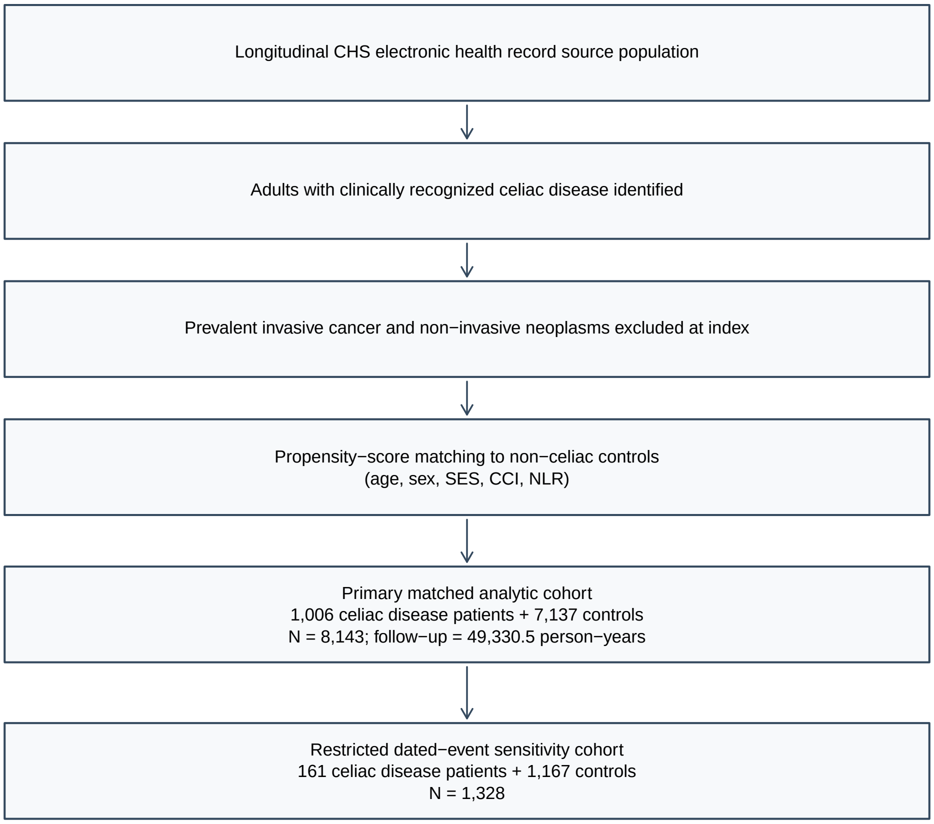

Figure S1: Cohort construction and analytic flow.

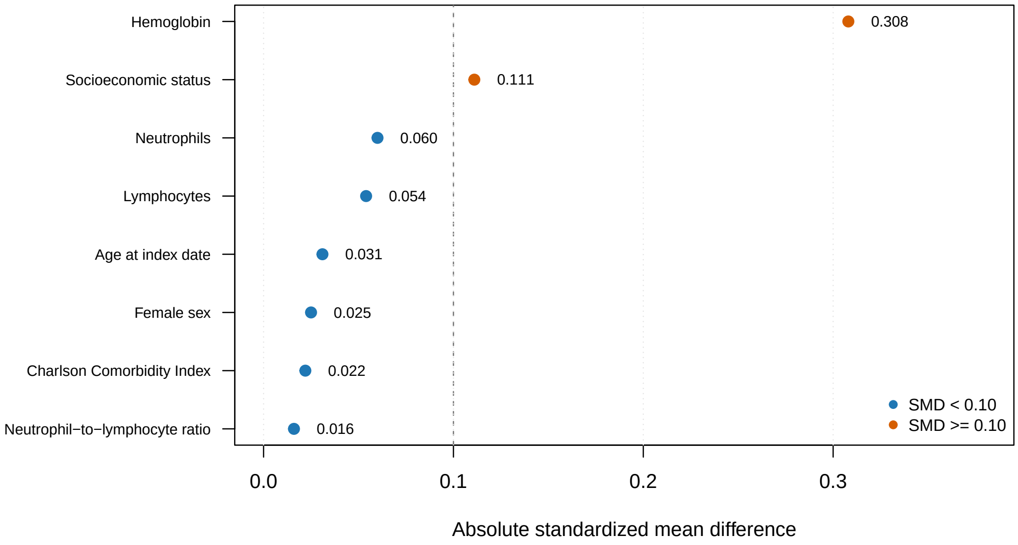

Figure S2: Standardized mean differences for variables shown in the primary matched cohort baseline table. Hemoglobin was shown descriptively and was not included in the matching algorithm.

Supplementary Tables

Supplementary Table S1: Full Multivariable Cox Regression Model for Hematological Malignancies

Table S1: Full multivariable Cox regression model for hematological malignancies (primary cohort)

**Variable**	**HR**	**95% CI**	**P-value**
Celiac disease	1.99	1.75–2.26	<0.001
Male sex	1.22	1.08–1.37	0.002
Socioeconomic status			
Low	1.15	0.91–1.44	0.239
Medium	0.90	0.74–1.09	0.282
Very Low	1.23	1.04–1.46	0.017
Very High	0.72	0.56–0.91	0.006
No data	0.74	0.56–0.97	0.027
Charlson Comorbidity Index	0.94	0.92–0.97	<0.001
Neutrophil-to-lymphocyte ratio	0.93	0.91–0.95	<0.001

Abbreviations: HR, hazard ratio; CI, confidence interval.
Model adjusted for all listed covariates with robust standard errors clustered by matched subclass.
Reference category for socioeconomic status was "High".

Supplementary Table S2: Full Multivariable Cox Regression Model for Lymphoma

Table S2: Full multivariable Cox regression model for lymphoma (primary cohort)

Variable	HR	95% CI	P-value
Celiac disease	1.90	1.61–2.25	<0.001
Male sex	1.30	1.12–1.50	<0.001
Socioeconomic status			
Low	1.21	0.92–1.57	0.169
Medium	1.12	0.89–1.40	0.324
Very Low	1.40	1.15–1.71	<0.001
Very High	0.67	0.51–0.88	0.004
No data	0.57	0.39–0.81	0.002
Charlson Comorbidity Index	0.94	0.91–0.96	<0.001
Neutrophil-to-lymphocyte ratio	0.98	0.96–0.99	0.010

Abbreviations: HR, hazard ratio; CI, confidence interval.
Model adjusted for all listed covariates with robust standard errors clustered by matched subclass.
Reference category for socioeconomic status was "High".

Supplementary Table S3: Full Multivariable Cox Regression Model for 1-Year Lag Sensitivity Analysis

Table S3: Full multivariable Cox regression model for 1-year lag sensitivity analysis (primary cohort)

Variable	HR	95% CI	P-value
Celiac disease	1.88	1.69–2.09	<0.001
Male sex	1.05	0.96–1.14	0.273
Socioeconomic status			
Low	0.93	0.78–1.13	0.479
Medium	0.94	0.80–1.11	0.488
Very Low	1.65	1.49–1.83	<0.001
Very High	0.94	0.82–1.09	0.407
No data	1.24	0.99–1.55	0.057
Charlson Comorbidity Index	1.04	1.03–1.06	<0.001
Neutrophil-to-lymphocyte ratio	0.98	0.97–0.99	<0.001

Abbreviations: HR, hazard ratio; CI, confidence interval.

Model adjusted for all listed covariates with robust standard errors clustered by matched subclass.

Number of events=4,623.

HRs are celiac disease versus matched controls.

Supplementary Table S4: Full Multivariable Cox Regression Model for 2-Year Lag Sensitivity Analysis

Table S4: Full multivariable Cox regression model for 2-year lag sensitivity analysis (primary cohort)

Variable	HR	95% CI	P-value
Celiac disease	1.89	1.69–2.11	<0.001
Male sex	1.09	1.00–1.19	0.060
Socioeconomic status			
Low	1.03	0.85–1.24	0.799
Medium	0.98	0.83–1.16	0.834
Very Low	1.80	1.62–2.01	<0.001
Very High	0.99	0.85–1.14	0.856
No data	1.35	1.07–1.70	0.011
Charlson Comorbidity Index	1.03	1.02–1.05	<0.001
Neutrophil-to-lymphocyte ratio	0.97	0.96–0.98	<0.001

Abbreviations: HR, hazard ratio; CI, confidence interval.
Model adjusted for all listed covariates with robust standard errors clustered by matched subclass.
Number of events=4,131.
HRs are celiac disease versus matched controls.

Supplementary Table S5: Cancer Identification and Exclusion Summary

Table S5: Cancer identification and exclusion summary

Processing Step	Count
Total diagnosis records screened	415,546
Non-malignant or non-invasive records excluded	171,302
Records remaining after exclusion	244,244

Excluded records comprised non-invasive neoplasms, benign conditions, and neoplasms of uncertain or unspecified behavior. The analytic cohort required valid temporal alignment of exposure, index date, and incident cancer outcome.

Supplementary Table S6: Cancer Subtype Detection Summary

Table S6: Cancer subtype detection summary from source diagnosis records

Cancer Subtype	Diagnosis Record Count
Lymphoma	28,766
Leukemia	8,824
Gastrointestinal cancer	28,794
Breast cancer	19,667
Lung cancer	13,799
Prostate cancer	22,741
Skin invasive	16,849
Genitourinary	31,948
Central nervous system	2,594
Head and neck	2,978

Counts are diagnosis records identified for each cancer subtype category.

Supplementary Table S7: Hemoglobin-Adjusted Cox Model (Dated-Event Cohort)

Table S7: Full multivariable Cox regression model with hemoglobin added, dated-event cohort

Variable	HR	95% CI	P-value
Celiac disease	1.61	1.26–2.05	<0.001
Male sex	1.01	0.84–1.21	0.921
Socioeconomic status			
Low	0.97	0.79–1.19	0.772
Medium	0.87	0.69–1.10	0.240
Very Low	1.30	0.92–1.84	0.140
Very High	0.85	0.65–1.12	0.242
No data	1.07	0.75–1.55	0.700
Charlson Comorbidity Index	1.07	1.05–1.09	<0.001
Neutrophil-to-lymphocyte ratio	1.00	0.99–1.01	0.930
Hemoglobin (per g/dL)	0.94	0.91–0.98	0.001

Abbreviations: HR, hazard ratio; CI, confidence interval.
Dated-event cohort restricted to records with non-missing hemoglobin (n=1,720). Reference category for socioeconomic status was "High". Robust standard errors clustered by matched subclass.

Supplementary Table S8: Fine–Gray Competing-Risk Model (Dated-Event Cohort)

Table S8: Fine–Gray subdistribution hazard model with death as a competing event, dated-event cohort

Variable	SHR	95% CI	P-value
Celiac disease	1.69	1.31–2.19	<0.001
Male sex	1.00	0.88–1.14	0.83
Socioeconomic status			
Low	1.00	0.84–1.19	1.00
Medium	0.95	0.80–1.13	0.58
Very Low	1.33	1.00–1.77	0.052
Very High	0.87	0.69–1.09	0.23
No data	1.15	0.84–1.57	0.38
Charlson Comorbidity Index	1.05	1.04–1.07	<0.001
Neutrophil-to-lymphocyte ratio	0.99	0.98–1.01	0.45

Abbreviations: SHR, subdistribution hazard ratio; CI, confidence interval.

Dated-event cohort (n=1,771). Death was treated as a competing event.

Reference category for socioeconomic status was "High".

Supplementary Table S9: High-Specificity Celiac Disease Exposure Analysis

Table S9: Celiac disease and all invasive cancer under a high-specificity exposure definition

Exposure definition	HR	95% CI	P-value
Dated-event cohort, all CD codes	1.68	1.31–2.14	<0.001
High-specificity CD (≥2 documentation fields)	1.68	1.31–2.14	<0.001

Abbreviations: HR, hazard ratio; CI, confidence interval.

All 161 CD patients met the ≥2-field criterion, so the high-specificity cohort was identical to the dated-event cohort. Model adjusted for sex, socioeconomic status, Charlson Comorbidity Index, and neutrophil-to-lymphocyte ratio.

Supplementary Table S10: Age-at-Index Subgroup Estimates

Table S10: Celiac disease and all invasive cancer stratified by age at index date

Age-at-index stratum	Celiac disease HR
Younger (<63.9 years)	1.37
Older (≥63.9 years)	2.12

Abbreviations: HR, hazard ratio.
Split at the cohort median age at index (63.9 years). Exploratory point estimates adjusted for sex, socioeconomic status, Charlson Comorbidity Index, and neutrophil-to-lymphocyte ratio; per-stratum events were few, so confidence intervals are not reported.

Supplementary Table S11: T-cell Lymphoma and EATL-proxy Diagnosis Descriptions

Table S11: Lymphoma diagnosis descriptions classified as peripheral T-cell lymphoma or EATL-proxy

Diagnosis description (verbatim EHR text)	Classification
Peripheral T-cell lymphoma, intra-abdominal lymph nodes	T-cell; EATL-proxy
Peripheral T-cell lymphoma, unspecified site, extranodal and solid-organ sites	T-cell; EATL-proxy
Other malignant lymphomas, unspecified site, extranodal and solid-organ sites	EATL-proxy (gut-oriented)

EATL, enteropathy-associated T-cell lymphoma.
